# Cumulative Incidence, Risk Factors, and Overall Survival of Disease Recurrence after Curative Resection of Stage II–III Colorectal Cancer: A Population-based Study

**DOI:** 10.1158/2767-9764.CRC-23-0512

**Published:** 2024-02-29

**Authors:** Tara C. Boute, Hidde Swartjes, Marjolein J.E. Greuter, Marloes A.G. Elferink, Rik van Eekelen, Geraldine R. Vink, Johannes H.W. de Wilt, Veerle M.H. Coupé

**Affiliations:** 1 Department of Epidemiology and Data Science, Amsterdam Public Health Research Institute, Amsterdam UMC, location Vrije Universiteit, Amsterdam, the Netherlands.; 2 Department of Surgery, Radboud University Medical Center, Nijmegen, the Netherlands.; 3 Department of Research and Development, Netherlands Comprehensive Cancer Organisation, Utrecht, the Netherlands.; 4 Department of Medical Oncology, University Medical Center Utrecht, Utrecht University, Utrecht, the Netherlands.

## Abstract

Real-world data are necessitated to counsel patients about the risk for recurrent disease after curative treatment of colorectal cancer. This study provided a population-based overview of the epidemiology of recurrent disease in patients with surgically resected stage II/III colorectal cancer.

Patients diagnosed with stage II/III primary colorectal cancer between July and December 2015 were selected from the Netherlands Cancer Registry (*N* = 3,762). Cumulative incidence of recurrent disease was estimated, and multivariable competing risk regression was used to identify risk factors for recurrent disease in patients with primary colon and rectal cancer. Moreover, overall survival (OS) after diagnosis of recurrent colorectal cancer was estimated.

Median clinical follow-up was 58 months (Q_1_–Q_3_: 22–62). Five-year cumulative incidence of recurrent disease was 21.6% [95% confidence interval (CI): 20.0–23.2] and 30.0% (95% CI: 28.3–33.5) for patients with primary colon and rectal cancer, respectively. Stage III disease and incomplete resection margin in patients with primary colon cancer and extramural vascular invasion in patients with primary rectal cancer were strongly (HR ≥ 2) associated with recurrent disease. Median OS of patients with distant, locoregional, or the synchronous combination of distant and locoregional recurrent disease was 29, 27, and 13 months, respectively (*P* < 0.001). Patients with distant recurrences limited to liver or lung showed a median OS of 46 and 48 months, respectively. The incidence of recurrent disease was higher in patients with rectal cancer than in patients with colon cancer, predominantly due to higher rates of distant recurrences. OS after recurrent disease was impaired, but subgroups of patients diagnosed with recurrent disease limited to one site showed statistically significantly longer OS.

**Significance:**

Population-based data on recurrent colorectal cancer are rare, but crucial for counseling patients and their physicians. This large nationwide, population-based study provides an up-to-date overview of the epidemiology of recurrent disease in patients with stage II and III primary colon and rectal cancer treated with surgical resection.

## Introduction

Colorectal cancer is a disease with a major global burden (1). Almost 4 out of 5 patients with colorectal cancer are diagnosed when the disease has not yet spread to distant organs or sites (2, 3). The majority of patients with colorectal cancer is therefore eligible for curative surgical resection. Because of earlier and improved diagnosis, more effective treatment of the primary tumor and metastases, the overall survival (OS) rate of patients with colorectal cancer has improved notably over the last decades (2, 4, 5).

Approximately 20%–30% of patients with stage II and III colorectal cancer experience a recurrence within the first 5 years after resection (6–8) with the majority of recurrences diagnosed within the first 2 years after treatment (9). Previous studies indicated that patients with a higher age, advanced tumor stage, nodal spread, presence of lymphatic or vascular invasion, and poor tumor differentiation at diagnosis of the primary colorectal cancer were at increased risk for recurrent colorectal cancer (10–12). Disease recurrence obviously has a major impact on OS in these patients (8).

However, when curative-intent treatment can be given in patients with recurrent disease, OS greatly increases. For example, trial patients treated with curative-intent treatment for liver only recurrence(s) have shown a median OS of over 60 months (13). Localization, pattern, and tumor load of the recurrent colorectal cancer are the main factors dictating treatment and potentially influencing OS (14–16). Besides, disease stage of the primary tumor and molecular factors are also strongly associated with OS after diagnosis of recurrent disease (8).

Accurate and up-to-date epidemiologic information about disease recurrence is of importance to inform patients on their recurrence risk during colorectal cancer follow-up. Population-based, nationwide studies are best suited to provide such estimates, because they are less prone to selection bias and thus allow for comprehensive interpretation. However, population-based studies on recurrent colorectal cancer are rare, as registration of diagnoses of recurrent colorectal cancer requires extensive effort.

The Netherlands Cancer Registry has recently collected recurrence data of patients with primary stage II and III colorectal cancer with 5 years follow-up after resection. Using these data, the aim of this study was to assess the cumulative incidence of recurrent disease and factors associated with diagnosis of recurrent disease in patients with stage II and III primary colon or rectal cancer who underwent surgical resection, as well as to assess OS after diagnosis of recurrent colorectal cancer.

## Materials and Methods

### Data Collection

Patient and tumor data were obtained from the Netherlands Cancer Registry (NCR), managed by the Netherlands Comprehensive Cancer Organisation (IKNL). Trained data managers extracted data from electronic patient files in all Dutch hospitals, after notification of newly diagnosed patients through the automated national pathological archive (PALGA). All patients diagnosed with stage II and III colorectal cancer between July 1 and December 31 of 2015 were included. The additional data collection about recurrent disease was performed in 2021. No data were available about the treatment of recurrent colorectal cancer. Annual linkage of the NCR with the Dutch personal records database on February 1, 2022 provided the vital status. The Privacy Review Board of the NCR approved this study.

### Study Design and Population

In this retrospective, population-based cohort study, the following patients were excluded: patients in whom the primary tumor was not treated with a surgical resection, patients with an appendiceal, overlapping or unspecified localization of the primary tumor, patients with neuroendocrine tumor morphology and patients without follow-up data (Supplementary Fig. S1). In patients diagnosed with more than one synchronous primary colorectal tumor, the characteristics of the tumor with the highest disease stage were used.

### Definitions of Diagnoses and Outcomes

The 7th edition of the Unio Internationale Contra Cancrum TNM (tumor–node–metastasis) classification was used as reference for the clinical and pathologic tumor stage (17). Pathologic stage was used, but in patients treated with neoadjuvant treatment and in patients in whom pathologic staging was missing, clinical stage was used.

The standard of the International Classification of Disease-Oncology (ICD-O-3) was used as reference for the tumor localization and morphology (18). Tumor localization was categorised as colon (C18.0, C18.2-C18.7) or rectum (C19.9, C20.9). Morphology was grouped into non-mucinous adenocarcinoma (8140–8389), mucinous adenocarcinoma (8470, 8480, 8481), signet cell carcinoma (8490), and other morphologies. Charlson comorbidity index was used to report comorbidities (19). The American Society of Anesthesiologists (ASA) physical status classification system is used to categorize patients based on their overall health status before surgery (20).

Recurrent disease was defined as any recurrence of colorectal cancer which presented after resection of the primary tumor. A locoregional recurrence (LRR) was defined as recurrent colorectal cancer near the site of the resection, or in lymph nodes defined as regional lymph nodes by the TNM classification. A distant recurrence (DR) was defined as recurrent colorectal cancer not meeting the definition of LRR, and localization was registered according to the third edition of the ICD-O-3.

Clinical follow-up time was measured from the date of resection of the primary tumor until detection of the first recurrence of colorectal cancer, death or last clinical contact with the hospital. Follow-up until vital status was measured from date of resection of the primary tumor until date of vital status (i.e., deceased or alive) registered in the Dutch personal records database.

### Statistical Analyses

The primary outcomes of this study were cumulative incidence of recurrent colorectal cancer, factors associated with diagnosis of recurrent colorectal cancer and OS after recurrent colorectal cancer. First, an overview of the population characteristics was provided using descriptive statistics. Second, the cumulative incidence of recurrent disease at 1, 3, and 5 years after primary tumor resection was estimated for patients with primary colon and rectal cancer, stratified for primary disease stage. The cumulative incidence was also estimated separately for LRR only, DR only or both LRR and DR. Cumulative incidence was adjusted to account for the competing risk of death, and each function was compared using Gray test (21).

Third, univariable and multivariable associations between patient- and tumor-related factors and recurrent disease were estimated using competing risk regression according to the subdistribution hazards approach (22). Again, death was considered a competing risk and the outcomes were presented for primary colon and rectal cancer. Age, ASA physical status, morphology, differentiation grade, the number of assessed lymph nodes, and resection margin were categorized to enhance clinical interpretation or to improve statistical power due to an otherwise small group size. All factors included in the univariable model were included in the multivariable model, except for emergency resection because of possible collinearity with bowel obstruction at presentation.

Fourth, OS after diagnosis of recurrent colorectal cancer was estimated using the Kaplan–Meier method, and differences between survival curves in localization and pattern were tested using the log-rank test.

### Missing Data and Software

To reduce bias due to missing data, multiple imputation with chained equations was used (23). Additional information on the handling of missing data by multiple imputation is presented in Supplementary Data S1. Sensitivity analyses without multiple imputation were performed for the risk factors and were reported in Supplementary Table S5.

All statistical analyses were performed using R version 4.1.3 with the “cmprsk” and “survival” package. The “mice” package was used for the multiple imputation. *P* < 0.05 was regarded as statistically significant.

### Data Availability

The data that support the findings of this study are available from the IKNL. Restrictions apply to the availability of these data, which were used under license for this study. Data are available from the corresponding author, T.C. Boute, with the permission of IKNL.

## Results

### Patient and Tumor Characteristics

In total, 3,762 patients were included in this study (Supplementary Fig. S1). Median age of all included patients was 69 years (Q_1_–Q_3_: 63–77) and 54.8% was male. The primary tumor was located in the colon in 70.9% of patients, and in the rectum in 29.1% of patients. Patient and tumor characteristics, stratified between patients with primary colon and rectal cancer, are presented in Table 1. The majority of patients with primary colon cancer were diagnosed with stage II disease (52.5%), while stage III disease was more common in patients with primary rectal cancer (70.6%). Patients with primary colon cancer with stage II and III disease were treated with adjuvant chemotherapy in 8.1% and 63.6% of cases, respectively (Supplementary Table S1). Patients with primary rectal cancer with stage II and III disease were treated with neoadjuvant (chemo)radiation in 52.5% and 79.0% of cases, respectively.

**TABLE 1:** Patient and tumor characteristics of patients with primary colon and rectal cancer

Characteristics	Colon cancer *N* (%) or median (Q_1_–Q_3_)	Rectal cancer *N* (%) or median (Q_1_–Q_3_)	Total *N* (%) or median (Q1–Q3)
Total number of patients	2,668	1,094	3,762
Sex			
Male	1,377 (51.6)	686 (62.7)	2,063 (54.8)
Female	1,291 (48.4)	408 (37.3)	1,699 (45.2)
Age (years)	71 (65–78)	67 (61–74)	69 (63–77)
ASA physical status			
I	390 (14.6)	236 (21.6)	626 (16.6)
II	1,437 (53.9)	609 (55.7)	2,046 (54.4)
III	573 (21.5)	158 (14.4)	731 (19.4)
IV/V	37 (1.3)	7 (0.6)	44 (1.1)
Unknown/missing	231 (8.7)	84 (7.7)	315 (8.4)
No. of comorbidities			
0	1,261 (47.3)	586 (53.6)	1,847 (49.1)
1	834 (31.3)	284 (26.0)	1,118 (29.7)
≥2	389 (14.6)	136 (12.4)	525 (14.0)
Unknown/missing	184 (6.9)	88 (8.0)	272 (7.2)
Disease stage			
II	1,401 (52.5)	322 (29.4)	1,723 (45.8)
III	1,267 (47.5)	772 (70.6)	2,039 (54.2)
Pathologic tumor stage			
(y)pT0	1 (0.0)	95 (8.7)	96 (2.6)
(y)pT1	56 (2.1)	64 (5.9)	120 (3.2)
(y)pT2	133 (5.0)	238 (21.8)	371 (9.9)
(y)pT3	1,984 (74.4)	638 (58.3)	2,622 (69.7)
(y)pT4	492 (18.4)	54 (4.9)	546 (14.5)
Unknown/missing	2 (0.1)	5 (0.4)	7 (0.2)
Pathologic nodal stage			
(y)pN0	1,413 (53.0)	647 (59.1)	2,060 (54.8)
(y)pN1	882 (33.0)	346 (31.6)	1,228 (32.6)
(y)pN2	373 (14.0)	101 (9.3)	474 (12.6)
Resection margin			
R0	2,590 (97.1)	1,040 (95.1)	3,630 (96.5)
R1	44 (1.6)	45 (4.1)	89 (2.4)
R2	8 (0.3)	3 (0.3)	11 (0.3)
Unknown/missing	26 (1.0)	6 (0.5)	32 (0.9)
Morphology			
Non-mucinous adenocarcinoma	2,305 (86.4)	1,029 (94.1)	3,334 (88.6)
Mucinous adenocarcinoma	311 (11.7)	60 (5.5)	371 (9.9)
Signet cell carcinoma	32 (1.2)	3 (0.3)	35 (0.9)
Other	20 (0.7)	2 (0.2)	22 (0.6)
Differentiation grade			
Good differentiation	67 (2.5)	25 (2.2)	92 (2.4)
Moderate differentiation	1,996 (74.8)	909 (83.1)	2,905 (77.2)
Poor differentiation	323 (12.1)	63 (5.8)	386 (10.3)
No differentiation	4 (0.1)	1 (0.1)	5 (0.1)
Unknown/missing	278 (10.5)	96 (8.8)	374 (9.9)
Vascular invasion			
No vascular invasion	2,212 (82.9)	851 (77.8)	3,063 (81.4)
IMVI	92 (3.4)	47 (4.3)	139 (3.7)
EMVI	327 (12.3)	114 (10.4)	441 (11.7)
Unknown/missing	37 (1.4)	82 (7.5)	119 (3.2)
Lymphatic invasion			
No	2,050 (76.8)	856 (78.2)	2,906 (77.2)
Yes	524 (19.6)	128 (11.7)	652 (17.3)
Unknown/missing	94 (3.6)	110 (10.1)	204 (5.4)
No. of assessed lymph nodes	18 (14–25)	15 (11–20)	17 (13–24)
Bowel obstruction at presentation			
No	2,273 (85.2)	1,058 (96.7)	3,331 (88.5)
Yes	363 (13.6)	26 (2.4)	389 (10.3)
Unknown/missing	32 (1.2)	10 (0.9)	42 (1.1)
Emergency resection			
No	2,320 (87.0)	1,065 (97.3)	3,385 (90.0)
Yes	340 (12.7)	26 (2.4)	366 (9.7)
Unknown/missing	8 (0.3)	3 (0.3)	11 (0.3)
Surgical approach			
Laparoscopic	1,620 (60.7)	834 (76.3)	2,454 (65.2)
Open	1,048 (39.3)	252 (23.0)	1,300 (34.6)
Unknown/missing	0 (0.0)	8 (0.7)	8 (0.2)
Tumor perforation			
No	2,416 (90.6)	1,007 (92.0)	3,423 (91.0)
Yes	124 (4.6)	37 (3.4)	161 (4.3)
Unknown/missing	128 (4.8)	50 (4.6)	178 (4.7)
Anastomotic leakage			
No	2,275 (85.3)	593 (54.2)	2,868 (76.2)
Yes	144 (5.4)	83 (7.6)	227 (6.0)
No anastomosis	216 (8.1)	389 (35.6)	605 (16.1)
Unknown/missing	33 (1.2)	29 (2.7)	62 (1.6)

Abbreviations: ASA, American Society of Anaesthesiologists; R0, resection margin of ≥1 mm; R1, resection margin of 0–1 mm; R2, macroscopically incomplete resection margin; IMVI, intramural vascular invasion; EMVI, extramural vascular invasion.

### Cumulative Incidence of Recurrent Colorectal Cancer

Median clinical follow-up time was 57.5 months (Q_1_–Q_3_: 22.2–62.2). In total, 896 patients (23.8%) developed recurrent disease (primary colon cancer: *N* = 328; primary rectal cancer: *N* = 568). The median time to diagnosis of recurrent disease was 15.2 months (Q_1_–Q_3_: 8.1–27.6); 542 patients (14.4%) died during clinical follow-up due to other causes than recurrent disease. One-, 3-, and 5-year cumulative incidence estimates and absolute numbers of patients diagnosed with recurrent disease are included in Supplementary Table S2. Within 3 and 6 months after resection of the primary tumor, 40 (1.1%) and 127 patients (3.4%) were diagnosed with recurrent disease, respectively.

**FIGURE 1:**
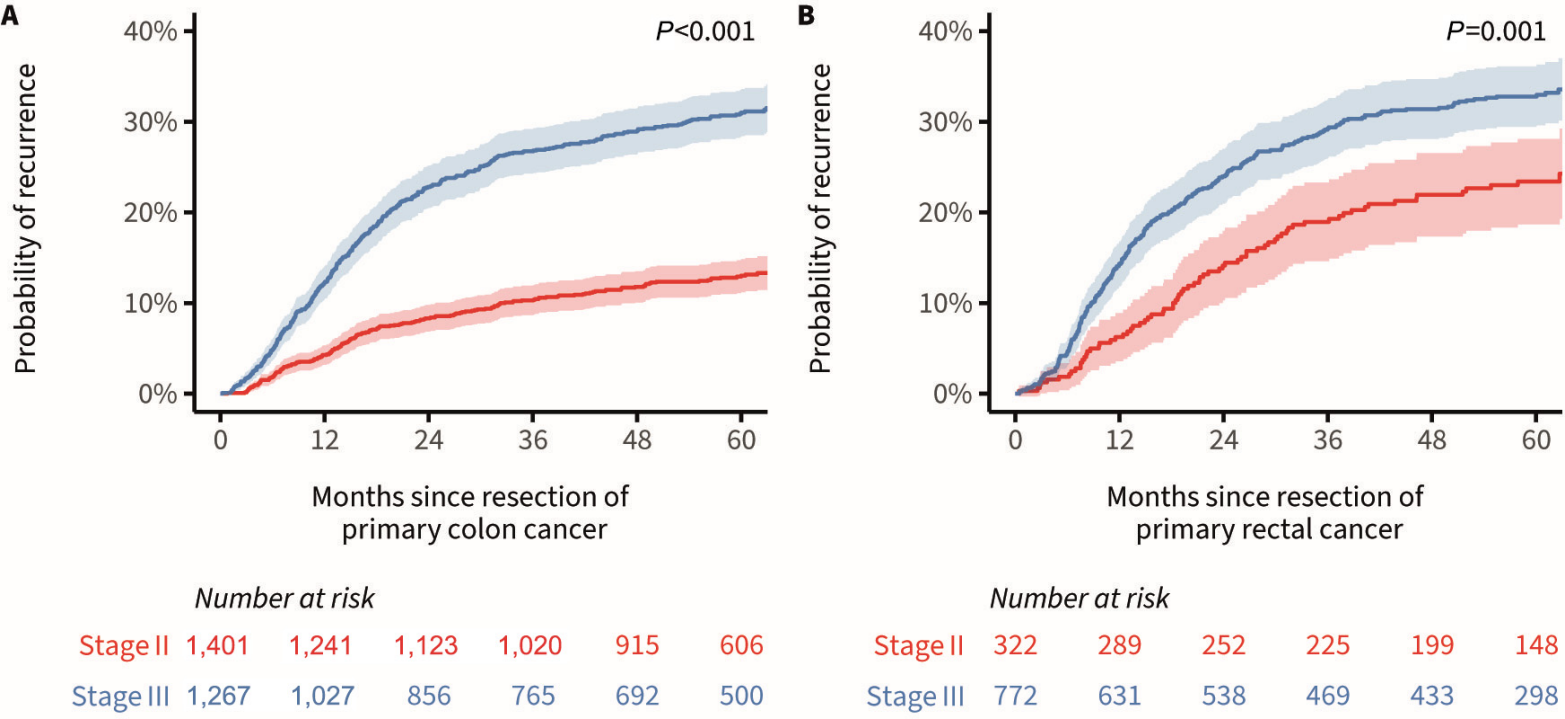
Cumulative incidence function for diagnosis of recurrent disease in patients with primary colon (**A**) and rectal cancer (**B**), stratified for primary disease stage and presented with the corresponding 95% confidence bands.

The 5-year cumulative incidence of recurrent disease in patients with primary colon cancer was 21.6% [95% confidence interval (CI): 20.0–23.2] overall, and 13.0% (95% CI: 11.2–14.8) for stage II and 30.9% (95% CI: 28.3–33.5) for patients with stage III primary colon cancer (Fig. 1A). In patients with primary rectal cancer, the 5-year cumulative incidence of recurrent disease was 30.0% (95% CI: 27.3–32.8) overall, and 23.4% (95% CI: 18.7–28.1) for stage II and 32.8% (95% CI: 29.4–36.1) for patients with stage III primary rectal cancer (Fig. 1B).

**FIGURE 2:**
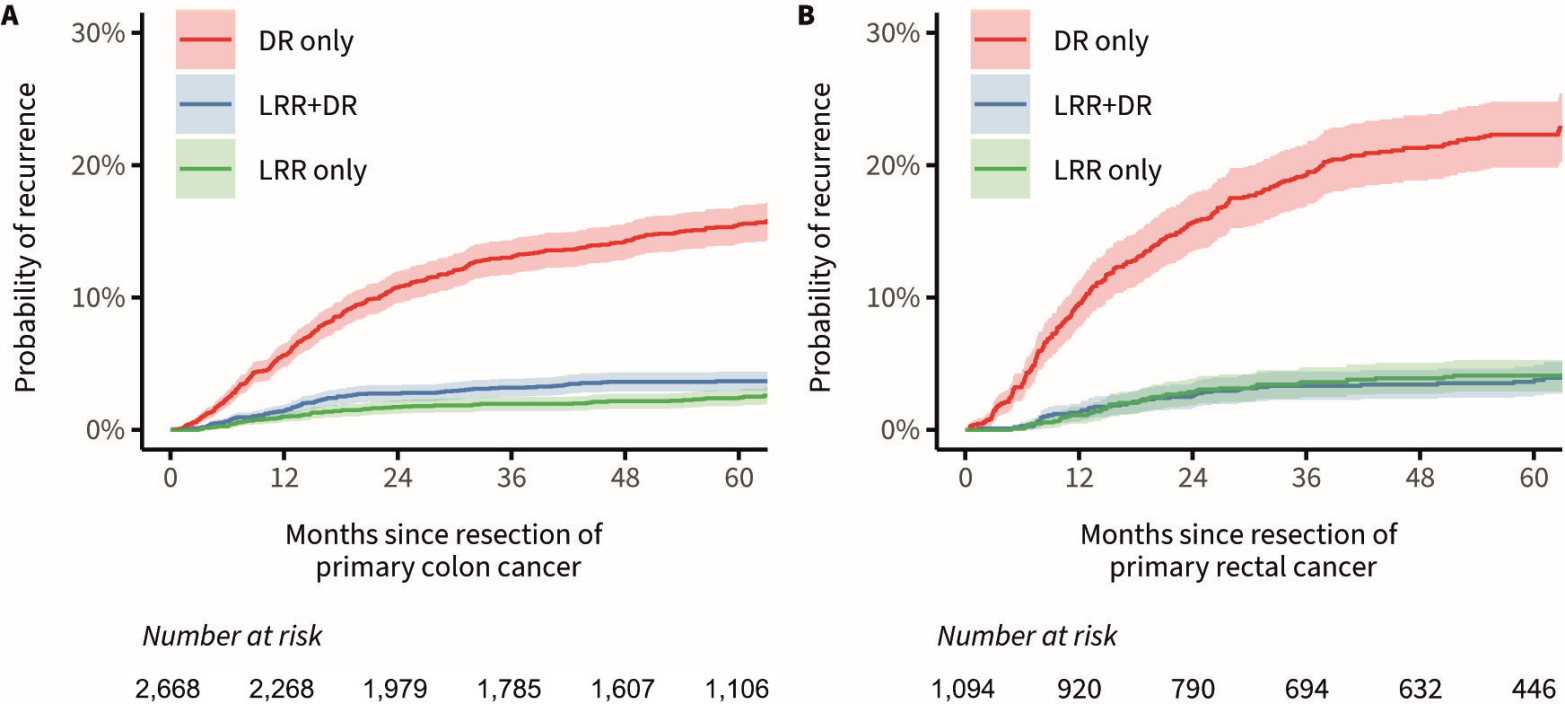
Cumulative incidence functions of distant recurrent disease (i.e., DR only), locoregional recurrent disease (i.e., LRR only) and the synchronous combination of LRR+DR in patients with primary colon (**A**) and rectal cancer (**B**), presented with the corresponding 95% confidence bands.

The 5-year cumulative incidence of distant recurrent disease (i.e., DR only) was 15.5% (95% CI: 14.1–16.9) in patients with primary colon cancer. The 5-year cumulative incidence of locoregional recurrent disease (i.e., LRR only) was 2.4% (95% CI: 1.8–3.0) in patients with primary colon cancer, and the 5-year cumulative incidence of the combination of LRR+DR was 3.7% (95% CI: 2.9–4.4) in this group (Fig. 2A). In patients with rectal cancer, the 5-year cumulative incidence of DR only was 22.3% (95% CI: 19.8–24.8), and the 5-year cumulative incidences of LRR only and LRR+DR were 4.0% (95% CI: 2.9–5.3) and 3.6% (95% CI: 2.5–4.7), respectively (Fig. 2B).

### Patient and Tumor Factors Associated with Diagnosis of Recurrent Disease in Patients with Primary Colon and Rectal Cancer

The results of the univariable and multivariable analyses for the risk of diagnosis with recurrent disease in patients with primary colon and rectal cancer are included in Supplementary Tables S3 and S4, respectively, along with 5-year cumulative incidence rates stratified for the included factors.

**FIGURE 3:**
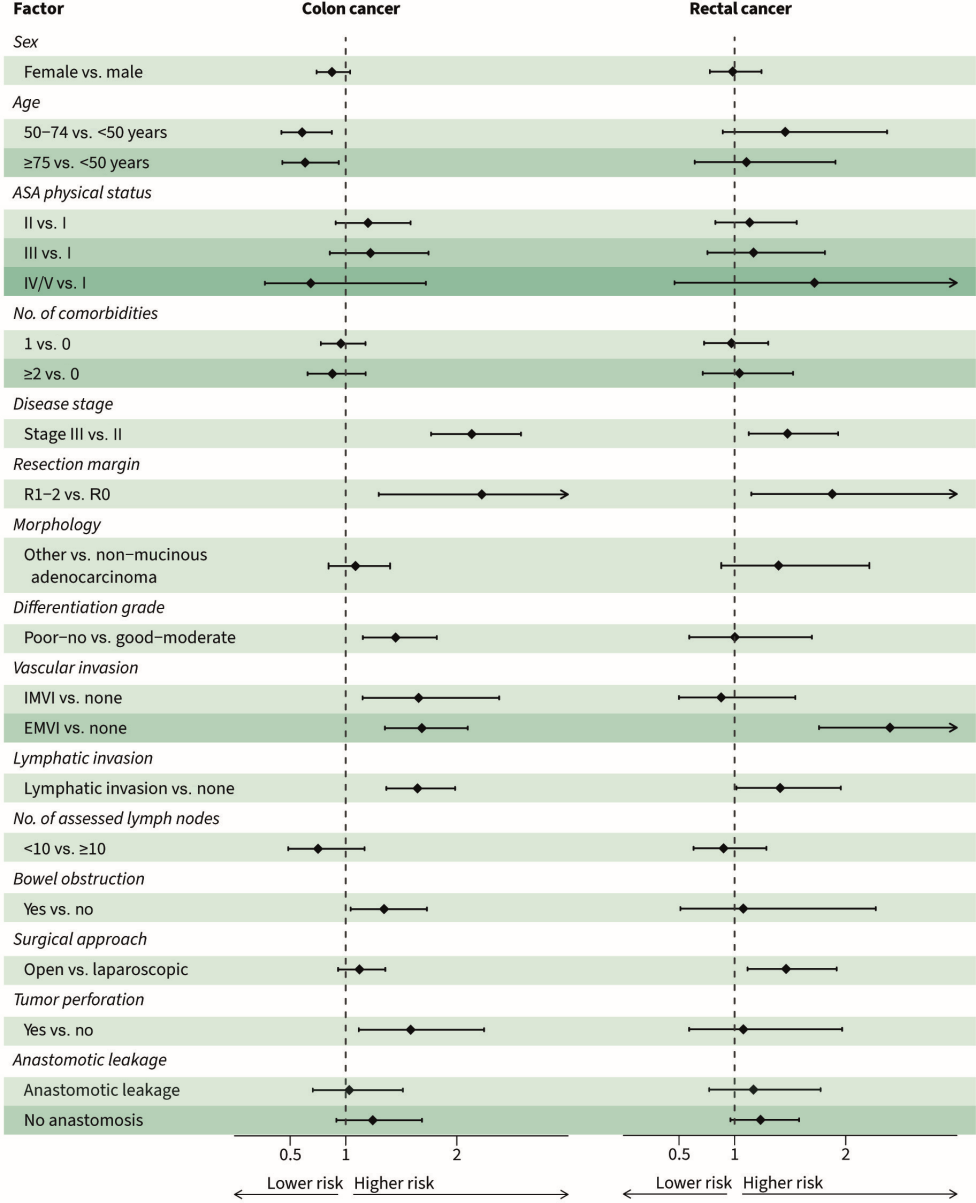
Forest plot of risk for diagnosis with recurrent disease in patients with primary colon and rectal cancer, based on multivariable competing risk regression models. HR; Hazard ratio. ASA; American Society of Anesthesiologists. R0; resection margin of ≥1 mm. R1; resection margin of 0–1 mm. R2; macroscopically incomplete resection margin. IMVI; intramural vascular invasion. EMVI; extramural vascular invasion.

In patients with primary colon cancer, stage III disease (HR = 2.1, 95% CI: 1.8–2.6), an incomplete resection margin (HR = 2.3, 95% CI: 1.3–4.0), poor or no tumor differentiation (HR = 1.5, 95% CI: 1.2–1.8), intramural vascular invasion (HR = 1.7, 95% CI: 1.2–2.5), extramural vascular invasion (HR = 1.7, 95% CI: 1.4–2.1), lymphatic invasion (HR = 1.6, 95% CI: 1.4–2.0), bowel obstruction at presentation (HR = 1.3, 95% CI: 1.0–1.7), and tumor perforation (HR = 1.6, 95% CI: 1.1–2.3) were statistically significantly associated with a higher risk for diagnosis of recurrent disease in the multivariable analysis (Fig. 3). Stage III disease (HR = 1.4, 95% CI: 1.1–1.9), an incomplete resection margin (HR = 2.0, 95% CI: 1.2–3.2), extramural vascular invasion (HR = 2.4, 95% CI: 1.8–3.3), and open surgical approach (HR = 1.5, 95% CI: 1.1–1.9) were statistically significantly associated with a higher risk for diagnosis of recurrent disease in patients with primary rectal cancer. Moreover, age ≥75 years was significantly associated with a lower risk for disease recurrence compared with age <65 years in patients with rectal cancer (HR = 0.7, 95% CI: 0.5–1.0).

The results of the multivariable analyses without multiple imputation are shown in Supplementary Table S5. In patients with colon cancer, age 65–74 years was strongly associated with a lower risk of recurrent disease in the analyses without multiple imputation (HR = 0.5, 95% CI: 0.3–0.9). For patients with rectum cancer, ASA score III (HR = 1.7, 95% CI: 1.0–2.7), resection margin (HR = 3.2, 95% CI: 1.9–5.6), and tumor perforation (HR = 1.8, 95% CI: 1.1–2.1) were stronger correlated to recurrence risk in the analyses without multiple imputation. Also, age 65–75 years was no longer associated with a lower recurrence risk (HR = 0.8, 95% CI: 0.6–1.0) compared with age <65 years, rather, it indicated a higher risk (HR = 1.2, 95% CI: 0.7–2.0).

### OS after Diagnosis of Recurrent Colorectal Cancer

Median follow-up time between diagnosis of recurrent colorectal cancer and vital status was 23.6 months (Q_1_–Q_3_: 9.8–43.5). Median OS of patients with any colorectal cancer recurrence was 25.5 months (95% CI: 23.3–27.4). No statistically significant difference in OS was found between patients with recurrent colorectal cancer with regard to primary cancer stage (median OS for primary stage III vs. stage II: 24.2 vs. 27.3 months, *P* = 0.077).

**FIGURE 4:**
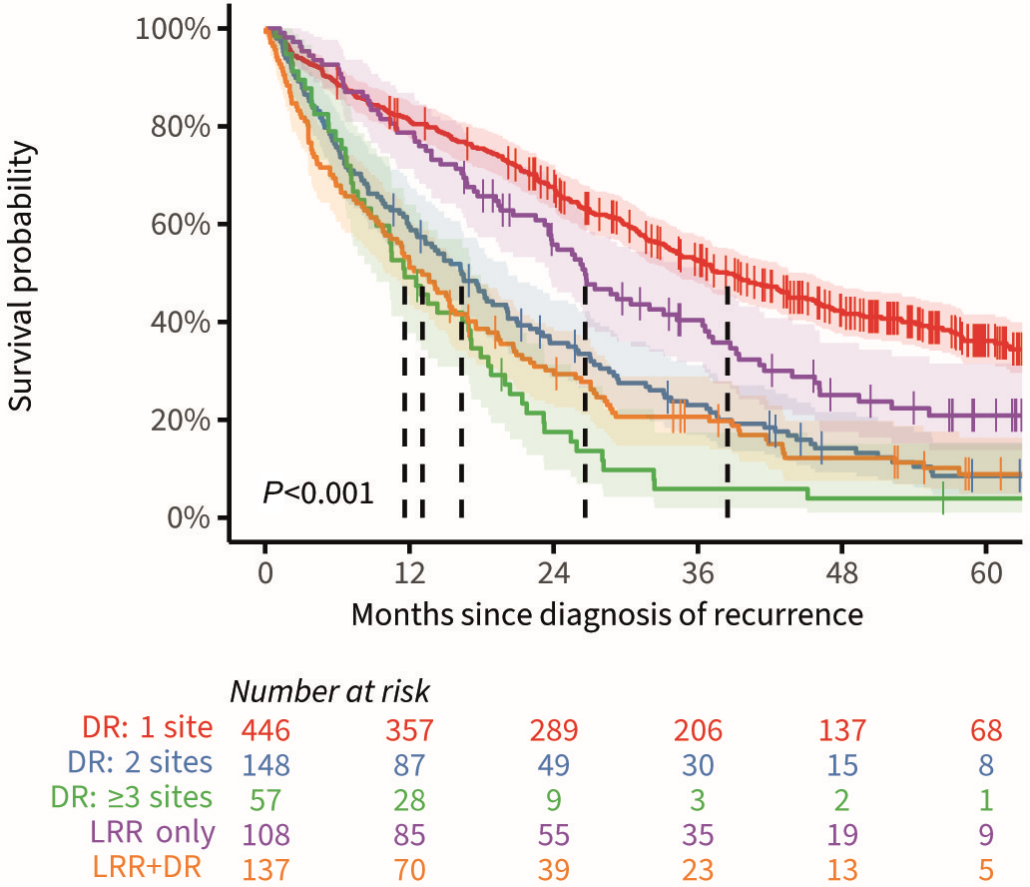
OS plot of patients diagnosed with recurrent colorectal cancer with corresponding 95% confidence bands, stratified for locoregionally recurrent colorectal cancer only (LRR), distantly recurrent colorectal cancer (DR) at one site, DR at two sites, DR at two or more sites, and the synchronous diagnosis of DR+LRR. Vertically dashed lines represent median OS.

Median OS of patients with DR only, LRR only and LRR+DR was 28.6 months (95% CI: 25.4–31.7), 26.6 months (95% CI: 23.6–36.6), and 13.1 months (95% CI: 10.0–16.8), respectively (*P* < 0.001). Median OS of DR only patients with metastases in one site, two sites, or three or more sites was 38.5 months (95% CI: 33.9–45.0), 16.3 months (95% CI: 12.6–20.2), and 11.6 months (95% CI: 8.8–17.1), respectively (*P* < 0.001, Fig. 4).

**FIGURE 5:**
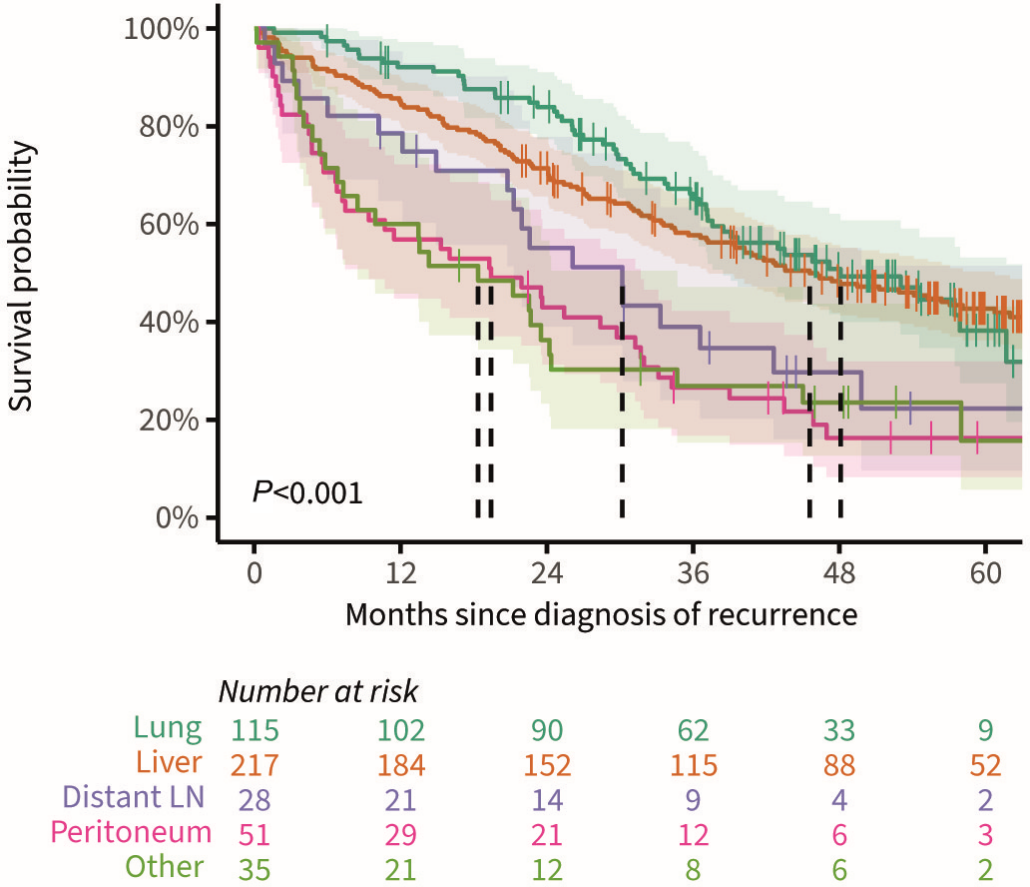
OS plot of patients diagnosed with distantly recurrent colorectal cancer at one site only with corresponding 95% confidence bands, stratified for the localization of the recurrence(s). Vertically dashed lines represent median OS. LN, lymph nodes.

OS of DR only patients with metastases in one site differed statistically significantly between the different localizations of the recurrences (*P* < 0.001, Fig. 5). Median OS of patients with isolated recurrent colorectal cancer located in the liver, lung, lymph nodes or peritoneum was 45.6 months (95% CI: 37.2–60.7), 48.1 months (95% CI: 38.5–upper limit not reached), 30.2 months (95% CI: 21.3–upper limit not reached), and 19.4 months (95% CI: 9.4–31.7), respectively (Fig. 5). For the heterogenous group of patients with distant metastases located in any other isolated site (*N* = 35), median OS was 18.4 months (95% CI: 8.5–34.7).

Patients diagnosed with recurrent colorectal cancer in the first year after resection of the primary tumor showed an impaired OS compared with patients diagnosed with recurrent colorectal cancer after this first year (17.5 vs. 28.9 months, HR = 1.5, 95% CI: 1.3–1.7). The 1-, 3-, and 5-year OS statistics of all abovementioned subgroups are presented in Supplementary Table S6.

## Discussion

This study found a 5-year cumulative incidence of recurrent disease in surgically resected stage II and III primary colon cancer of 13.0% and 30.9%, respectively, in rectal cancer, these numbers were 23.4% and 32.8%, respectively. Patients with rectal cancer were more frequently diagnosed with DR only. Stage III disease and an incomplete resection margin were strongly associated (i.e., HR with ≥2) with diagnosis of recurrent disease in patients with primary colon cancer, while in rectal cancer extramural vascular invasion showed a strong association with recurrence. OS of patients diagnosed with recurrent colorectal cancer in more than one site was poor, while subgroups of patients diagnosed with recurrent colorectal cancer in the liver or lung only showed statistically significantly longer OS than patients with recurrent colorectal cancer in other sites.

Few population-based studies have assessed the cumulative incidence of recurrent disease in primary colon and rectal cancer patients, perhaps due to the additional effort needed to collect the necessary clinical follow-up data for the entire population. Nonetheless, the 5-year cumulative incidence of DR only estimated in the current study (primary colon cancer: 15.5%; primary rectal cancer: 22.3%) was comparable with rates found in other international studies (16%–22%; refs. 24–27). On the other hand, the 5-year cumulative incidences of LRR and LRR+DR are low in comparison with the (inter)national literature (28–31). This could be indicative of adequate presurgical assessment of resectability, as locoregional failure is clearly associated with incomplete resection margins and locally advanced tumors (28, 32, 33). Previous studies have been performed in the Netherlands using data from the first semester of 2015 (11, 14, 15), but were limited by the availability of 3 years of clinical follow-up data only. While these studies suggest a potential flattening of recurrence curves around the 3-year mark, the current study showed an additional 120 recurrences detected between year 3 and 5. This led to an increase in cumulative incidence from 20.5% in year 3 to 24.1% in year 5. Given that follow-up guidelines recommend a 5-year schedule, the current study presents a more comprehensive report of recurrence incidence.

The 3-year cumulative incidence of recurrent disease after surgical resection of stage II and III colon cancer in the current study (stage II: 10.3%; stage III: 26.7%) was very comparable with that of patients diagnosed in the first semester of 2015 (stage II: 12%–16%; stage III: 24%–31%; ref. 11). However, the incidence rates of recurrence for surgically treated stage II and III rectal cancer seemed lower for patients diagnosed in the second semester of 2015 (stage II: 19.0%; stage III: 29.3%) than in the first semester of 2015 (stage II: 24%; stage III: 38%). The most straightforward explanation for this difference lies in the classification of disease stage: Qaderi and colleagues used the pathologic TNM classification for all patients (11), regardless of neoadjuvant treatment. The current study reported the clinical instead of the pathologic TNM classification for patients treated with neoadjuvant treatment. Herewith, the results of the current study reflect the risk for recurrent disease in regard to the extent of the disease at presentation, and not after neoadjuvant treatment.

The cumulative incidence of recurrent disease increased rapidly in the first 2 years after resection of the primary colon or rectal cancer, but flattened between the third and fifth year after surgical resection. This finding validates the duration of the advised oncological follow-up for surgically resected colorectal cancer in Western countries (34–36), which is limited to 5 years after surgical resection. Other studies have demonstrated that the incidence of recurrent disease more than 5 years after surgical resection of primary colon or rectal cancer is exceptionally low (37–41).

Prognostic factors such as extramural vascular invasion, lymphatic invasion, but also tumor deposits and tumor budding are a focus of ongoing research because of their assumed ability to possibly enhance the TNM classification in regard to risk classification (42–44). Stage III disease and an incomplete resection margin showed the strongest associations with diagnosis of recurrent disease, but intramural vascular invasion, extramural vascular invasion and lymphatic invasion showed associations of substantial strength as well. Nonrandomized studies have been able to show an OS benefit for adjuvant chemotherapy in patients with stage II primary colon cancer with positive extramural vascular invasion (45) or lymphovascular invasion (46). However, pT4 tumors, <10 investigated lymph nodes, tumor perforation, obstruction, vascular invasion and poor-no tumor differentiation were all classifications for a high-risk stage II tumor, and consequentially and indication for adjuvant chemotherapy in the treatment guideline in the Netherlands in 2014. This guideline was revised in 2019, and based on various population-based studies, the classification of a high-risk stage II tumor was narrowed to pT4 only. Undoubtedly, adjuvant chemotherapy for patients with stage II colon cancer will remain controversial, as is illustrated by the practice variation in the Netherlands and the absent OS benefit on a population base (47). In patients with primary rectal cancer, extramural vascular invasion showed the strongest association with diagnosis of recurrent disease of all included factors, vastly stronger than lymphatic invasion. The presence of MRI-based extramural vascular invasion is currently implemented in the clinical management, and is an absolute indication for neoadjuvant chemoradiation or short-course radiotherapy (35, 48–50).

Notably, Qaderi and colleagues found a higher recurrent disease risk in patients with stage I–II colon cancer ages between 65 and 74 compared with those ages <65 years old (11). No such association was found in the current study in patients with stage II–III colon cancer. The most likely explanation for the difference in results between the two studies probably lies in the difference in the statistical models. The relationship between age and disease recurrence was adjusted for more confounding factors in the present model [21 vs. 14 degrees of freedom in current study versus Qaderi and colleagues (11)].

Five-year OS after diagnosis of any recurrence was estimated at 23.5%. Few studies have estimated the OS of patients with recurrent colorectal cancer, especially patients who were primarily diagnosed with stage II or III disease. The COLOFOL trial population consisted of stage II and III colorectal cancer only, and reported a 32% 5-year OS after recurrence detection for these patients (51). It is not unexpected to find a difference in OS between randomized clinical trial populations and nationwide populations, as participants in clinical trials are likely to be in better health.

The OS of patients diagnosed with recurrent colorectal cancer was clearly related to the pattern of the recurrence: patients diagnosed with LRR only or DR only in one site showed superior OS outcomes in comparison with patients diagnosed with LRR+DR or DR in two or more sites. In clinical practice, patients with synchronous or metachronous metastases limited to one site are treated with curative intent if local treatment of all metastases falls within the clinical possibilities (52). This treatment effect is likely the cause of the relatively long median OS for liver only (48.1 months) and lung only (45.6 months) recurrences estimated in this study. In limited cases, curative-intent treatment is given to patients with synchronously diagnosed LRR and DR (14, 15), or distant metastases in two or more sites (53). However, the majority of patients diagnosed with recurrent colorectal cancer in two or more locations are treated with palliative intent or best supportive care, (54) leading to median OS estimates in the current study of less than 18 months.

This study has several limitations. First, no recurrence data were available for the group of patients with stage I primary colorectal cancer who are generally also treated with surgical resection (i.e., pT2N0M0). Nonetheless, it is known that recurrent colorectal cancer in this group is rare (24). Second, no information was available about the number of recurrences present in each organ/site, about the occurrence of subsequent (re-)recurrences, nor about the treatment of recurrences. However, these treatment data are rarely reported in population-based studies, and generally only collected in clinical trials. These data would have been of value in the stratification of OS after diagnosis of recurrent colorectal cancer, especially in the comparison of different treatment strategies for the management of recurrent disease. Therefore, it would be preferable if future rounds of data collections about recurrent colorectal cancer for cancer registries would include data about treatment of recurrences, while it must be acknowledged that resources for additional data collection are limited. Also, drawing conclusions from such information would be challenging due to underlying mechanisms such as patient preferences and medical history, which could significantly influence treatment outcomes. Third, the nationwide colorectal cancer screening program in the Netherlands was started in 2014, although not yet fully operational in the second semester of 2015. This program consists of biennial faecal immunochemical testing for the population between 55 and 75 years. Only a small cohort of patients (∼10%) received screening in the second semester of 2015 (55), thus the effect is expected to be small. In several years’ time, a reassessment of the cumulative incidence, associated factors and OS in the population in which colorectal cancer screening is fully operational is needed.

## Conclusion

This retrospective, nationwide, population-based study provides an up-to-date overview of the epidemiology of recurrent disease in patients with stage II and III primary colon and rectal cancer treated with surgical resection. The 5-year cumulative incidence of recurrent disease was 13.0% and 30.9% for patients with stage II and III primary colon cancer, respectively, and 23.4% and 32.8% for patients with stage II and III primary rectal cancer, respectively. The majority of recurrent disease was localized distantly, especially in patients with primary rectal cancer. OS differed statistically significantly between the different patterns of recurrence, with patients with isolated metastasis in the liver or lung showing longer OS, while patients with recurrent colorectal cancer synchronously diagnosed in two or more sites had severely impaired OS outcomes.

## Supplementary Material

Supplementary Figure 1 Flowchart of exclusions

Supplementary Table 1 Treatment characteristics

Supplementary Text 1 Methodology for Multiple imputation

Supplementary Table 2 One-, three- and five-year cumulative incidence estimates of recurrent disease in primary colon and rectal cancer patients.

Supplementary Table 3 Univariable and multivariable competing risk regression output for the risk of recurrent disease in colon cancer patients

Supplementary Table 4 Univariable and multivariable competing risk regression output for the risk of recurrent disease in rectal cancer patients

Supplementary Table 5 Univariable and multivariable competing risk regression output for the risk of recurrent disease in colon cancer patients

Supplementary Table 6 One-, three- and five-year overall survival of patients with recurrent colorectal cancer
