# Supplementary figures and images for: Cumulative Incidence, Risk Factors, and Overall Survival of Disease Recurrence after Curative Resection of Stage II–III Colorectal Cancer: A Population-based Study
Source: Cancer Res Commun. 2024 Feb 29;4(2):607–16. doi: 10.1158/2767-9764.CRC-23-0512 (PMC10903299; doi:10.1158/2767-9764.CRC-23-0512)

**Supplementary Figure 1 – Flowchart of exclusions**


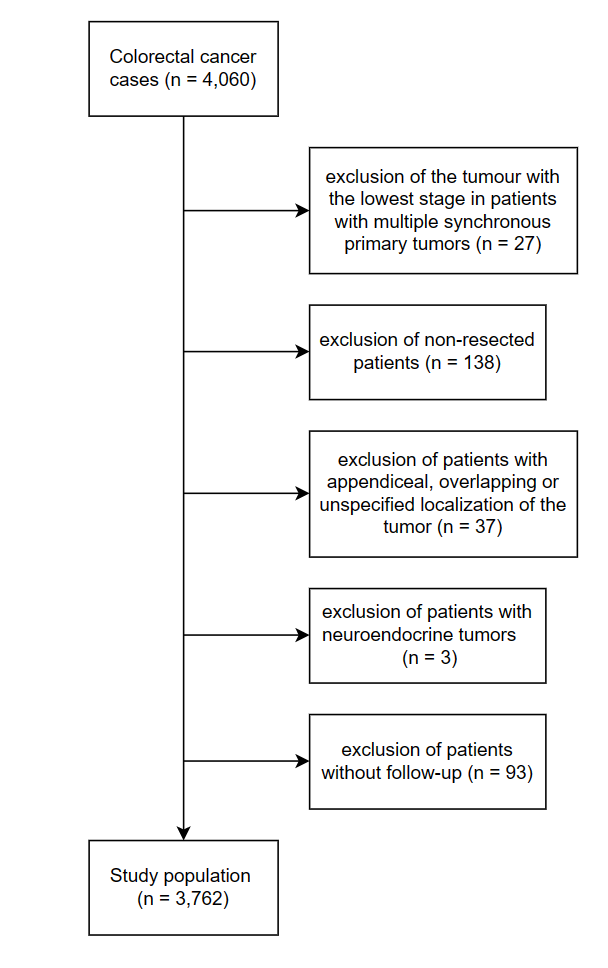

Supplement: Supplementary Figure 1 — Flowchart of exclusions [file crc-23-0512-s01.docx]
