# Supplementary material for: Cumulative Incidence, Risk Factors, and Overall Survival of Disease Recurrence after Curative Resection of Stage II–III Colorectal Cancer: A Population-based Study
Source: Cancer Res Commun. 2024 Feb 29;4(2):607–16. doi: 10.1158/2767-9764.CRC-23-0512 (PMC10903299; doi:10.1158/2767-9764.CRC-23-0512)
Supplement: Supplementary Table 1 — Treatment characteristics [file crc-23-0512-s02.docx]

**Supplementary Table 1 – Treatment characteristics**

|  | Stage II  N (%) | Stage III  N (%) |
| --- | --- | --- |
| Number of colon cancer patients | 1401 | 1267 |
| Treatment |  |  |
| Resection only | 1277 (91.1) | 446 (35.2) |
| Resection + adjuvant chemotherapy | 114 (8.1) | 806 (63.6) |
| Resection + other additional therapies | 10 (0.7) | 15 (1.2) |
|  |  |  |
| Number of rectal cancer patients | 322 | 772 |
| Treatment |  |  |
| Resection only | 149 (46.3) | 133 (17.2) |
| Resection + neoadjuvant radiotherapy | 125 (38.8) | 240 (31.1) |
| Resection + neoadjuvant chemoradiation | 44 (13.7) | 370 (47.9) |
| Resection + other additional therapies | 4 (1.2) | 29 (3.8) |
|  |  |  |
