# Supplementary material for: Cumulative Incidence, Risk Factors, and Overall Survival of Disease Recurrence after Curative Resection of Stage II–III Colorectal Cancer: A Population-based Study
Source: Cancer Res Commun. 2024 Feb 29;4(2):607–16. doi: 10.1158/2767-9764.CRC-23-0512 (PMC10903299; doi:10.1158/2767-9764.CRC-23-0512)
Supplement: Supplementary Text 1 — Methodology for Multiple imputation [file crc-23-0512-s03.docx]

**Supplementary Text 1 – Methodology for Multiple imputation**

To reduce potential bias due to missingness in the univariable and multivariable models, multiple imputation was used under the assumption that the data was missing at random (MAR) (1). Multivariate imputation by chained equations was performed using the “mice” package in R. The variables with missing values before multiple imputation were: comorbidities, bowel obstruction at presentation, tumour perforation, ASA performance score, differentiation grade, number of assessed lymph nodes, vascular invasion, lymphatic invasion, emergency surgery, surgical approach and anastomotic leakage. The following variables were included in the imputation model as predictors: age, sex, disease stage, resection margin, tumor site, morphology, , radiotherapy, chemotherapy, chemoradiation, recurrence and, vital status. We created 10 imputed datasets with 20 iterations each. The univariable and multivariable competing-risk regression analyses were performed on these datasets. Results were pooled subsequently according to Rubin’s rule (1).

*References:*

1. M.W. Heymans, I. Eekhout. Applied Missing Data Analysis With SPSS and (R)Studio. Vol. 1. Amsterdam; 2019.
