# Supplementary material for: Cumulative Incidence, Risk Factors, and Overall Survival of Disease Recurrence after Curative Resection of Stage II–III Colorectal Cancer: A Population-based Study
Source: Cancer Res Commun. 2024 Feb 29;4(2):607–16. doi: 10.1158/2767-9764.CRC-23-0512 (PMC10903299; doi:10.1158/2767-9764.CRC-23-0512)
Supplement: Supplementary Table 2 — One-, three- and five-year cumulative incidence estimates of recurrent disease in primary colon and rectal cancer patients. [file crc-23-0512-s04.docx]

**Supplementary Table 2** – One-, three- and five-year cumulative incidence estimates of recurrent disease in primary colon and rectal cancer patients.

|  | 1-year CI in % (95%-CI) | N recurrence at 1-year | 3-year CI in % (95%-CI) | N recurrence at 3-year | 5-year CI in %  (95%-CI) | N recurrence at 5-year |
| --- | --- | --- | --- | --- | --- | --- |
|  |  |  |  |  |  |  |
| Primary colorectal cancer patients |  |  |  |  |  |  |
| Overall *(N at risk=3762)* | 9.2 (8.3–10.1) | 343 | 20.5 (19.2–21.8) | 757 | 24.1 (22.7–25.5) | 877 |
|  |  |  |  |  |  |  |
| Primary colon cancer patients |  |  |  |  |  |  |
| Overall *(N at risk=2668)* | 8.1 (7.0–9.1) | 213 | 18.1 (16.7–19.6) | 473 | 21.6 (20.0–23.2) | 554 |
| LRR only* | 1.0 (0.6–1.4) | 26 | 2.0 (1.4–2.5) | 51 | 2.4 (1.8–3.0) | 61 |
| LRR+DR* | 1.4 (1.0–1.9) | 38 | 3.2 (2.5–3.9) | 83 | 3.7 (2.9–4.4) | 95 |
| DR only* | 5.6 (4.7–6.5) | 149 | 13.0 (11.7–14.3) | 339 | 15.5 (14.1–16.9) | 398 |
| Stage II *(N at risk=1401)* | 4.3 (3.2–5.3) | 59 | 10.3 (8.7–11.9) | 140 | 13.0 (11.2–14.8) | 173 |
| Stage III *(N at risk=1267)* | 12.3 (10.5–14.1) | 154 | 26.7 (24.3–29.2) | 333 | 30.9 (28.3–33.5) | 381 |
|  |  |  |  |  |  |  |
| Primary rectal cancer patients |  |  |  |  |  |  |
| Overall *(N at risk=1094)* | 11.9 (10.0–13.9) | 130 | 26.2 (23.6–28.9) | 284 | 30.0 (27.3–32.8) | 323 |
| LRR only* | 1.1 (0.5–1.7) | 12 | 3.6 (2.5–4.7) | 39 | 4.0 (2.9­–5.3) | 44 |
| LRR+DR* | 1.3 (0.6–2.0) | 14 | 3.3 (2.3–4.4) | 36 | 3.6 (2.5–4.7) | 39 |
| DR only* | 9.6 (7.8–11.3) | 104 | 19.3 (16.9–21.7) | 209 | 22.3 (19.8­–24.8) | 240 |
| Stage II *(N at risk=322)* | 6.2 (3.6–8.9) | 20 | 19.0 (14.6–23.3) | 60 | 23.4 (18.7­–28.1) | 73 |
| Stage III *(N at risk=772)* | 14.3 (11.9–16.8) | 110 | 29.3 (26.0–32.5) | 224 | 32.8 (29.4­–36.1) | 250 |
|  |  |  |  |  |  |  |

CI; cumulative incidence. 95%-CI; 95% confidence interval. LRR; locoregional recurrence. DR; distant recurrence. *No number at risk, because estimate is a dependent variable.
