# Supplementary material for: Cumulative Incidence, Risk Factors, and Overall Survival of Disease Recurrence after Curative Resection of Stage II–III Colorectal Cancer: A Population-based Study
Source: Cancer Res Commun. 2024 Feb 29;4(2):607–16. doi: 10.1158/2767-9764.CRC-23-0512 (PMC10903299; doi:10.1158/2767-9764.CRC-23-0512)
Supplement: Supplementary Table 3 — Univariable and multivariable competing risk regression output for the risk of recurrent disease in colon cancer patients [file crc-23-0512-s05.docx]

**Supplementary Table 3 –** Univariable and multivariable competing risk regression output for the risk of recurrent disease in colon cancer patients

|  | 5-year estimate of CRC recurrence, % (95%-CI) | Univariable  HR (95%-CI),  N=1,812 | *P* | Multivariable  HR (95%-CI),  N=1,812 | *P* |
| --- | --- | --- | --- | --- | --- |
|  |  |  |  |  |  |
| Sex |  |  |  |  |  |
| Male | 22.5 (20.2–24.7) | reference |  | reference |  |
| Female | 20.6 (18.3–22.8) | 0.9 (0.8–1.1) | 0.304 | 0.9 (0.7–1.0) | 0.133 |
| Age |  |  |  |  |  |
| <65 years | 23.1 (19.8–26.4) | reference |  | reference |  |
| 65-74 years | 21.1 (18.6–23.6) | 0.9 (0.7–1.1) | 0.367 | 0.9 (0.8–1.2) | 0.614 |
| ≥75 years | 21.2 (18.5–23.9) | 0.9 (0.7–1.1) | 0.284 | 0.9 (0.7–1.2) | 0.650 |
| ASA |  |  |  |  |  |
| I | 19.0 (15.2–22.8) | reference |  | reference |  |
| II | 22.1 (20.1–24.2) | 1.2 (0.9–1.5) | 0.160 | 1.2 (0.9–1.5) | 0.226 |
| III | 22.4 (19.1–25.8) | 1.2 (0.9–1.6) | 0.162 | 1.2 (0.9–1.7) | 0.294 |
| IV/V | 17.7 (5.7–29.9) | 1.0 (0.4–2.2) | 0.994 | 0.6 (0.3–1.5) | 0.309 |
| Number of comorbidities |  |  |  |  |  |
| 0 | 22.2 (19.9–24.4) | reference |  | reference |  |
| 1 | 21.4 (18.6–24.1) | 1.0 (0.8–1.2) | 0.634 | 0.9 (0.8–1.2) | 0.600 |
| ≥2 | 20.0 (16.1–24.0) | 0.9 (0.7–1.2) | 0.403 | 0.9 (0.7–1.2) | 0.489 |
| Disease stage |  |  |  |  |  |
| II | 13.0 (11.2–14.8) | reference |  | reference |  |
| III | 30.9 (28.3–33.5) | **2.7 (2.3**–**3.2)** | **<0.001** | **2.1 (1.8**–**2.6)** | **<0.001** |
| Resection margin |  |  |  |  |  |
| R0 | 21.1 (19.5–22.7) | reference |  | reference |  |
| R1-2 | 46.1 (32.3–59.8) | **3.1 (2.6**–**3.5)** | **<0.001** | **2.3 (1.3**–**4.0)** | **0.002** |
| Morphology |  |  |  |  |  |
| Non-mucinous adenocarcinoma | 21.5 (19.8–23.2) | reference |  | reference |  |
| Other | 22.0 (17.6–26.4) | 1.1 (0.8–1.3) | 0.672 | 1.1 (0.8–1.4) | 0.535 |
| Differentiation grade |  |  |  |  |  |
| Good-moderate differentiation | 19.9 (18.2–21.6) | reference |  | reference |  |
| Poor-no differentiation | 31.1 (26.5–35.8) | **1.8 (1.7**–**1.9)** | **<0.001** | **1.5 (1.2**–**1.8)** | **<0.001** |
| Vascular invasion |  |  |  |  |  |
| None | 18.7 (17.0–20.3) | reference |  | reference |  |
| IMVI | 35.1 (25.2–45.1) | **2.1 (1.5**–**3.1)** | **<0.001** | **1.7 (1.2–2.5)** | **0.003** |
| EMVI | 37.0 (31.8–42.3) | **2.4 (2.2**–**2.5)** | **<0.001** | **1.7 (1.4**–**2.1)** | **<0.001** |
| Lymphatic invasion |  |  |  |  |  |
| None | 17.7 (16.0–19.4) | reference |  | reference |  |
| Lymphatic invasion | 36.5 (32.4–40.6) | **2.3 (2.2**–**2.5)** | **<0.001** | **1.6 (1.4**–**2.0)** | **<0.001** |
| Amount of assessed lymph nodes |  |  |  |  |  |
| ≥10 lymph nodes | 21.7 (20.0–23.3) | reference |  | reference |  |
| <10 lymph nodes | 19.7 (12.4–26.9) | 0.9 (0.8–1.0) | 0.630 | 0.7 (0.5–1.2) | 0.190 |
| Bowel obstruction at presentation |  |  |  |  |  |
| No | 19.7 (18.1–21.4) | reference |  | reference |  |
| Yes | 33.3 (28.3–38.3) | **1.9 (1.7**–**2.0)** | **<0.001** | **1.3 (1.0**–**1.7)** | **0.022** |
| Emergency resection |  |  |  |  |  |
| No | 20.4 (18.7–22.1) | reference |  |  |  |
| Yes | 29.8 (24.8–34.8) | **1.6 (1.5**–**1.7)** | **<0.001** | *NR* |  |
| Surgical approach |  |  |  |  |  |
| Laparoscopic | 19.0 (17.1–21.0) | reference |  | reference |  |
| Open | 25.5 (22.8–28.2) | **1.5 (1.4**–**1.5)** | **<0.001** | 1.1 (0.9–1.4) | 0.184 |
| Tumour perforation |  |  |  |  |  |
| No | 21.0 (19.4–22.6) | reference |  | reference |  |
| Yes | 33.0 (24.9–41.2) | **1.9 (1.8**–**2.1)** | **<0.001** | **1.6 (1.1**–**2.3)** | **0.007** |
| Anastomotic leakage |  |  |  |  |  |
| No | 20.9 (19.2–22.6) | reference |  | reference |  |
| Yes | 21.0 (14.3–27.8) | 1.0 (0.7–1.5) | 0.965 | 1.1 (0.7–1.6) | 0.712 |
| No anastomosis | 28.7 (22.6–34.8) | **1.6 (1.2–2.0)** | **<0.001** | 1.2 (0.9–1.7) | 0.134 |

P-values <0.05 were regarded as statistically significant. HR; hazard ratio. 95%-CI; 95% confidence interval. NR; not reported. ASA; American Society of Anesthesiologists. R0; resection margin of ≥1 mm. R1; resection margin of 0-1 mm. R2; macroscopically incomplete resection margin. IMVI; intramural vascular invasion. EMVI; extramural vascular invasion.
