# Supplementary material for: Cumulative Incidence, Risk Factors, and Overall Survival of Disease Recurrence after Curative Resection of Stage II–III Colorectal Cancer: A Population-based Study
Source: Cancer Res Commun. 2024 Feb 29;4(2):607–16. doi: 10.1158/2767-9764.CRC-23-0512 (PMC10903299; doi:10.1158/2767-9764.CRC-23-0512)
Supplement: Supplementary Table 4 — Univariable and multivariable competing risk regression output for the risk of recurrent disease in rectal cancer patients [file crc-23-0512-s06.docx]

**Supplementary Table 4 –** Univariable and multivariable competing risk regression output for the risk of recurrent disease in rectal cancer patients

|  | 5-year estimate of CRC recurrence, % (95%-CI) | Univariable  HR (95%-CI),  N=1,094 | *P* | Multivariable  HR (95%-CI),  N=1,094 | *P* |
| --- | --- | --- | --- | --- | --- |
|  |  |  |  |  |  |
| Sex |  |  |  |  |  |
| Male | 30.5 (27.1–34.0) | reference |  | reference |  |
| Female | 29.1 (24.7–33.6) | 1.1 (0.7–1.2) | 0.465 | 1.0 (0.8–1.2) | 0.741 |
| Age |  |  |  |  |  |
| <65 years | 33.0 (28.5–37.5) | reference |  | reference |  |
| 65-74 years | 28.1 (23.7–32.4) | 0.8 (0.6–1.0) | 0.107 | 0.8 (0.6–1.0) | 0.070 |
| ≥75 years | 28.0 (22.3–33.7) | 0.8 (0.6–1.1) | **0.173** | **0.7 (0.5–1.0)** | **0.041** |
| ASA |  |  |  |  |  |
| I | 27.7 (22.0–33.3) | reference |  | reference |  |
| II | 29.8 (26.2–33.3) | 1.1 (0.8–1.4) | 0.568 | 1.2 (0.9–1.6) | 0.246 |
| III | 33.3 (26.5–40.2) | 1.2. (0.9–1.7) | 0.266 | 1.3 (0.9–2.0) | 0.218 |
| IV/V | * | 2.5 (0.8–7.7) | 0.105 | 1.9 (0.6–6.4) | 0.314 |
| Number of comorbidities |  |  |  |  |  |
| 0 | 30.0 (26.4–33.6) | reference |  | reference |  |
| 1 | 29.2 (24.1–34.2) | 1.0 (0.871.2) | 0.716 | 1.0 (0.7–1.5) | 0.756 |
| ≥2 | 32.1 (24.2–39.9) | 1.1 (0.8–1.5) | 0.706 | 1.1 (0.7–1.5) | 0.775 |
| Disease stage |  |  |  |  |  |
| II | 23.4 (18.7–28.1) | reference |  | reference |  |
| III | 32.8 (29.4–36.1) | **1.6 (1.2**–**2.0)** | **<0.001** | **1.4 (1.1**–**1.9)** | **0.009** |
| Resection margin |  |  |  |  |  |
| R0 | 28.9 (26.1–31.7) | reference |  | reference |  |
| R1-2 | 53.9 (39.6–68.2) | **2.5 (2.2**–**2.8)** | **<0.001** | **2.0 (1.2**–**3.2)** | **0.006** |
| Morphology |  |  |  |  |  |
| Non-mucinous adenocarcinoma | 29.8 (26.9 –32.6) | reference |  | reference |  |
| Other | 33.9 (22.3–45.6) | 1.2 (0.8–1.9) | 0.419 | 1.3 (0.8–2.1) | 0.247 |
| Differentiation grade |  |  |  |  |  |
| Good-moderate differentiation | 29.5 (26.7–32.4) | reference |  | reference |  |
| Poor-no differentiation | 36.6 (26.0–47.3) | **1.4 (1.2**–**1.6)** | 0.070 | 1.1 (0.7–1.8) | 0.560 |
| Vascular invasion |  |  |  |  |  |
| None | 26.8 (23.9–29.7) | reference |  | reference |  |
| IMVI | 29.1 (15.8–42.3) | 1.1 (0.6–1.9) | 0.742 | 0.9 (0.5–1.6) | 0.696 |
| EMVI | 55.4 (46.4–64.5) | **2.6 (1.9**–**3.4)** | **<0.001** | **2.4 (1.8**–**3.3)** | **<0.001** |
| Lymphatic invasion |  |  |  |  |  |
| None | 28.7 (25.8–31.7) | reference |  | reference |  |
| Lymphatic invasion | 38.5 (30.4–46.6) | **1.5(1.4**–**1.7)** | **<0.001** | 1.4 (0.9–1.9) | 0.096 |
| Amount of assessed lymph nodes |  |  |  |  |  |
| ≥10 lymph nodes | 30.1 (27.1–33.0) | reference |  | reference |  |
| <10 lymph nodes | 29.5 (21.6–37.4) | 1.0 (0.9–1.1) | 0.840 | 0.9 (0.6–1.3) | 0.501 |
| Bowel obstruction at presentation |  |  |  |  |  |
| No | 29.6 (26.9–32.4) | reference |  | reference |  |
| Yes | 44.9 (25.6–64.1) | **1.7 (1.4**–**2.1)** | **<0.001** | 1.2 (0.6–2.4) | 0.703 |
| Emergency resection |  |  |  |  |  |
| No | 29.8 (27.0–32.6) | reference |  |  |  |
| Yes | 38.9 (19.6–58.2) | **1.3 (1.1**–**1.5)** | 0.430 | *NR* |  |
| Surgical approach |  |  |  |  |  |
| Laparoscopic | 27.0 (23.9–30.0) | reference |  | reference |  |
| Open | 40.1 (34.0–46.2) | **1.6 (1.5**–**1.7)** | **<0.001** | **1.4 (1.1**–**1.9)** | **0.007** |
| Tumour perforation |  |  |  |  |  |
| No | 29.3 (26.6–32.1) | reference |  | reference |  |
| Yes | 48.5 (32.2–64.8) | **1.8 (1.5**–**2.1)** | **<0.001** | 1.0 (0.5–1.8) | 0.966 |
| Anastomotic leakage |  |  |  |  |  |
| No | 27.1 (23.6–30.7) | reference |  | reference |  |
| Yes | 33.3 (23.1–43.5) | 1.3 (0.9–1.9) | 0.292 | 1.2 (0.8–1.8) | 0.363 |
| No anastomosis | 33.8 (29.1–38.5) | **1.3 (1.0–1.7)** | **0.020** | 1.2 (0.9–1.5) | 0.141 |

P-values <0.05 were regarded as statistically significant. HR; hazard ratio. 95%-CI; 95% confidence interval. NR; not reported. ASA; American Society of Anesthesiologists. R0; resection margin of ≥1 mm. R1; resection margin of 0-1 mm. R2; macroscopically incomplete resection margin. IMVI; intramural vascular invasion. EMVI; extramural vascular invasion. *No events, sample size (N<10).
