# Supplementary material for: Cumulative Incidence, Risk Factors, and Overall Survival of Disease Recurrence after Curative Resection of Stage II–III Colorectal Cancer: A Population-based Study
Source: Cancer Res Commun. 2024 Feb 29;4(2):607–16. doi: 10.1158/2767-9764.CRC-23-0512 (PMC10903299; doi:10.1158/2767-9764.CRC-23-0512)
Supplement: Supplementary Table 5 — Univariable and multivariable competing risk regression output for the risk of recurrent disease in colon cancer patients [file crc-23-0512-s07.docx]

**Supplementary Table 5 - Univariable and multivariable competing risk regression output for the risk of recurrent disease in colon cancer patients**

|  | **multivariable model**  **with multiple imputation**  **colon**  **HR (95%-CI),**  *N=2,668* | **multivariable model**  **complete case analysis colon**  **HR (95%-CI),**  *N=1,812* | **multivariable model**  **with multiple imputation**  **rectum**  **HR (95%-CI),**  *N=1,094* | **multivariable model**  **complete case analysis rectum**  **HR (95%-CI),**  *N=715* |
| --- | --- | --- | --- | --- |
|  |  |  |  |  |
| Sex |  |  |  |  |
| Male | reference | reference | reference | reference |
| Female | 0.9 (0.7–1.0) | 0.9 (0.7–1.1) | 1.0 (0.8–1.2) | 0.9 (0.7–1.3) |
| Age |  |  |  |  |
| <65 years | reference | reference | reference | reference |
| 65-74 years | 0.9 (0.8–1.2) | **0.5 (0.3–0.9)** | 0.8 (0.6–1.0) | 1.2 (0.7–2.0) |
| ≥75 years | 0.9 (0.7–1.2) | 0.6 (0.4–1.0) | **0.7 (0.5–1.0)** | 0.7 (0.4–1.3) |
| ASA |  |  |  |  |
| I | reference | reference | reference | reference |
| II | 1.2 (0.9–1.5) | 1.1 (0.8–1.5) | 1.2 (0.9–1.6) | 1.2 (0.8–1.7) |
| III | 1.2 (0.9–1.7) | 1.1 (0.8–1.5) | 1.3 (0.9–2.0) | **1.7 (1.0–2.7)** |
| IV/V | 0.6 (0.3–1.5) | 0.7 (0.3–2.1) | 1.9 (0.6–6.4) | 2.4 (0.5–12.1) |
| Number of comorbidities |  |  |  |  |
| 0 | reference | reference | reference | reference |
| 1 | 0.9 (0.8–1.2) | 0.9 (0.7–1.2) | 1.0 (0.7–1.5) | 1.0 (0.7–1.3) |
| ≥2 | 0.9 (0.7–1.2) | 1.0 (0.7–1.4) | 1.1 (0.7–1.5) | 1.1 (0.7–1.6) |
| Disease stage |  |  |  |  |
| II | reference | reference | reference | reference |
| III | **2.1 (1.8**–**2.6)** | **2.1 (1.7**–**2.6)** | **1.4 (1.1**–**1.9)** | **1.6 (1.2**–**2.2)** |
| Resection margin |  |  |  |  |
| R0 | reference | reference | reference | reference |
| R1-2 | **2.3 (1.3**–**4.0)** | **2.0 (1.0**–**3.9)** | **2.0 (1.2**–**3.2)** | **3.2 (1.9**–**5.6)** |
| Morphology |  |  |  |  |
| Non-mucinous adenocarcinoma | reference | reference | reference | reference |
| Other | 1.1 (0.8–1.4) | 1.1 (0.7–1.7) | 1.3 (0.8–2.1) | 0.7 (0.2–1.5) |
| Differentiation grade |  |  |  |  |
| Good-moderate differentiation | reference | reference | reference | reference |
| Poor-no differentiation | **1.5 (1.2**–**1.8)** | 1.3 (1.0–1.7) | 1.1 (0.7–1.8) | 1.1 (0.6–1.9) |
| Vascular invasion |  |  |  |  |
| None | reference | reference | reference | reference |
| IMVI | **1.7 (1.2–2.5)** | **2.1 (1.4–3.1)** | 0.9 (0.5–1.6) | 0.7 (0.3–1.5) |
| EMVI | **1.7 (1.4**–**2.1)** | **1.6 (1.2**–**2.1)** | **2.4 (1.8**–**3.3)** | **2.6 (1.8**–**3.7)** |
| Lymphatic invasion |  |  |  |  |
| None | reference | reference | reference | reference |
| Lymphatic invasion | **1.6 (1.4**–**2.0)** | **1.6 (1.3**–**2.0)** | 1.4 (0.9–1.9) | 1.4 (0.9–2.0) |
| Amount of assessed lymph nodes |  |  |  |  |
| ≥10 lymph nodes | reference | reference | reference | reference |
| <10 lymph nodes | 0.7 (0.5–1.2) | 0.8 (0.5–1.3) | 0.9 (0.6–1.3) | 0.9 (0.6–1.3) |
| Bowel obstruction at presentation |  |  |  |  |
| No | reference | reference | reference | reference |
| Yes | **1.3 (1.0**–**1.7)** | **1.6 (1.2**–**2.4)** | 1.2 (0.6–2.4) | 1.0 (0.3–3.2) |
| Emergency resection |  |  |  |  |
| No |  |  |  |  |
| Yes | *NR* | *NR* | *NR* | *NR* |
| Surgical approach |  |  |  |  |
| Laparoscopic | reference | reference | reference | reference |
| Open | 1.1 (0.9–1.4) | 1.0 (0.8–1.3) | **1.4 (1.1**–**1.9)** | **1.5 (1.1**–**2.1)** |
| Tumour perforation |  |  |  |  |
| No | reference | reference | reference | reference |
| Yes | **1.6 (1.1**–**2.3)** | 1.5 (0.9–2.4) | 1.0 (0.5–1.8) | **1.8 (1.1–2.1)** |
| Anastomotic leakage |  |  |  |  |
| No | reference | reference | reference | reference |
| Yes | 1.1 (0.7–1.6) | 0.9 (0.6–1.5) | 1.2 (0.8–1.8) | 0.9 (0.5–1.6) |
| No anastomosis | 1.2 (0.9–1.7) | 1.3 (0.9–1.8) | 1.2 (0.9–1.5) | 1.0 (0.7–1.4) |
