# Supplementary material for: Cumulative Incidence, Risk Factors, and Overall Survival of Disease Recurrence after Curative Resection of Stage II–III Colorectal Cancer: A Population-based Study
Source: Cancer Res Commun. 2024 Feb 29;4(2):607–16. doi: 10.1158/2767-9764.CRC-23-0512 (PMC10903299; doi:10.1158/2767-9764.CRC-23-0512)
Supplement: Supplementary Table 6 — One-, three- and five-year overall survival of patients with recurrent colorectal cancer [file crc-23-0512-s08.docx]

**Supplementary Table 6 –** One-, three- and five-year overall survival of patients with recurrent colorectal cancer

| Pattern of recurrence | 1-year OS in % (95%-CI) | 3-year OS in % (95%-CI) | 5-year OS in % (95%-CI) |
| --- | --- | --- | --- |
|  |  |  |  |
| Any recurrence (N at risk=896) | 70.6 (67.7–73.7) | 38.4 (35.2–41.8) | 23.5 (20.6–26.8) |
| *Recurrence <1 year after resection (N at risk=553)* | 60.9 (56.0–66.3) | 29.4 (25.0–34.7) | 17.8 (14.2–22.3) |
| *Recurrence ≥1 year after resection (N at risk=343)* | 76.6 (73.2–80.2) | 44.1 (40.0–48.7) | 27.1 (22.9–32.0) |
|  |  |  |  |
| LRR only (N at risk=108) | 78.7 (71.3–86.8) | 40.4 (31.9–51.2) | 20.8 (13.8–31.4) |
| LRR+DR (N at risk=137) | 51.1 (43.3–60.2) | 20.6 (14.7–28.8) | 8.9 (4.8–28.8) |
| DR only (N at risk=651) | 73.4 (70.1–76.9) | 41.8 (38.0–45.8) | 26.9 (23.4–31.0) |
| *Located in 1 site (N at risk=446)* | 81.1 (77.6–84.8) | 52.5 (47.9–57.4) | 36.1 (31.5–41.5) |
| Liver only (N at risk=115) | 92.1 (87.3–97.2) | 65.1 (56.6–75.0) | 38.2 (27.5–53.1) |
| Lung only (N at risk=217) | 84.4 (80.1–89.7) | 57.7 (51.4–67.8) | 42.7 (36.3–50.3) |
| Distant lymph nodes only (N at risk=28) | 78.6 (64.8–95.3) | 39.0 (24.0–63.4) | 22.3 (9.6–51.5) |
| Peritoneum (N at risk=51) | 56.9 (44.8–72.2) | 26.6 (16.8–42.2) | 16.3 (8.3–31.9) |
| Other (N at risk=35) | 60.0 (45.8–78.6) | 26.9 (15.3–47.1) | 15.7 (5.7–43.2) |
| *Located in 2 sites (N at risk=148)* | 59.4 (52.0–67.9) | 23.0 (17.0–31.2) | 8.5 (4.7–15.3) |
| *Located in >2 sites (N at risk=57)* | 49.1 (37.7–64.0) | 5.8 (2.0–17.4) | 3.9 (1.0–15.0) |
|  |  |  |  |

OS; overall survival. 95%-CI; 95% confidence interval. LRR; locoregional recurrence. DR; distant recurrence
